# Paradoxical Cerebral Fat Embolism in Revision Hip Surgery

**DOI:** 10.1155/2014/140757

**Published:** 2014-08-11

**Authors:** Nicolás S. Piuzzi, Gerardo Zanotti, Fernando M. Comba, Martin A. Buttaro, Francisco Piccaluga

**Affiliations:** Hip Surgery Unit “Sir John Charnley”, Institute of Orthopaedics “Carlos E. Ottolenghi”, Italian Hospital of Buenos Aires, Peron 4190, 1181 Buenos Aires, Argentina

## Abstract

The incidence of clinical fat embolism syndrome (FES) is low (<1%) whilst fat embolism (FE) of marrow fat appears to occur more often (Mellor and Soni (2001)). Paradoxical brain FE may occur in patients undergoing hip orthopedic surgery who have an undocumented patent foramen ovale (PFO). We report a case of an eighty-year-old male patient, who underwent a scheduled revision hip surgery suffering a paradoxical cerebral FE.

## 1. Introduction

Fat embolism (FE) is defined as the presence of fat within the circulatory system, with or without clinical impact [1]. This entity may occur following long bone fractures, intramedullary nailing, and arthroplasty and may lead to coma or death [2–4]. Fat emboli might pass into the systemic circulation in the presence of venous to arterial (v-a) circulation shunt, a patent foramen ovale (PFO) being the most common cause [5].

Fat embolism syndrome (FES) is characterized by a set of respiratory, neurological, cutaneous, and hematological symptoms that may establish gradually or suddenly [6].

We report a case of a patient who sustained a paradoxical cerebral FE during revision hip surgery.

## 2. Case Report

An eighty-year-old male patient with multiple comorbidities (hypertension, dyslipidemia, hypothyroidism, myocardial reperfusion surgery 20 years ago, and type 2 second-degree AV block) underwent a scheduled revision hip surgery due to aseptic acetabular loosening.

Surgery was performed, under hypotensive epidural anesthesia, in lateral decubitus position using a posterolateral approach. Acetabular component was revised with a trabecular metal cup while the femoral component was done with a cement-cement technique. There were no abnormal intraoperative findings, particularly no decrease of oxygen saturation, no circulatory instability, normal heart rate, and no drop in arterial blood pressure (measured by noninvasive blood pressure).

Having completed the surgical procedure, the patient was transferred to the anesthesia recovery room, where fifteen minutes after arrival evolved stuporous with sensory impairment associated with left faciobrachiocrural hemiplegia. Immediately, tracheal intubation, mechanical respiratory assistance, and hemodynamic stabilization were established.

**Figure 1:**
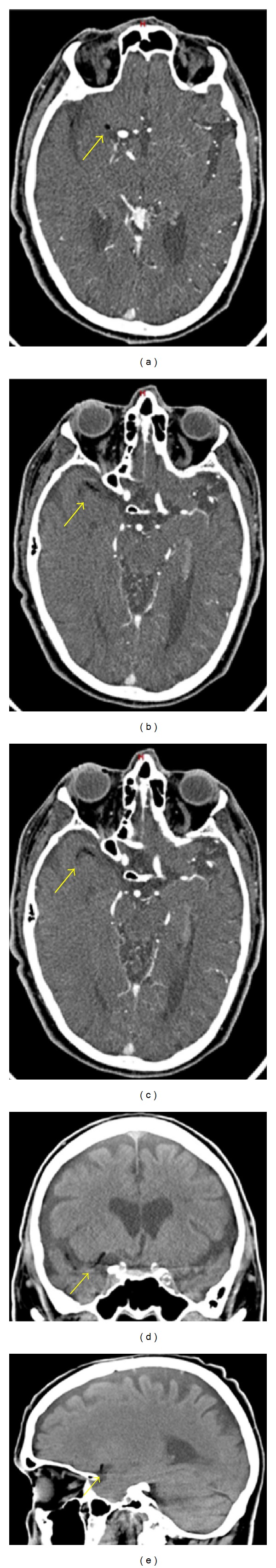
Computed tomography: axial ((a), (b), and (c)), coronal (d), and sagittal section images (e). Hypointense image is observed at the birth of the MCA (yellow arrow).

Within the first hour, a head computed angiotomography scan was conducted. This study showed a hypointense image, with the same intensity as fat tissue, at the middle cerebral artery (MCA) birth consistent with FE (Figure 1). With presumptive diagnosis of FE and being in 6-hour window from the time of the incision, we decided to perform an intra-arterial mechanical thrombolysis.

**Figure 2:**
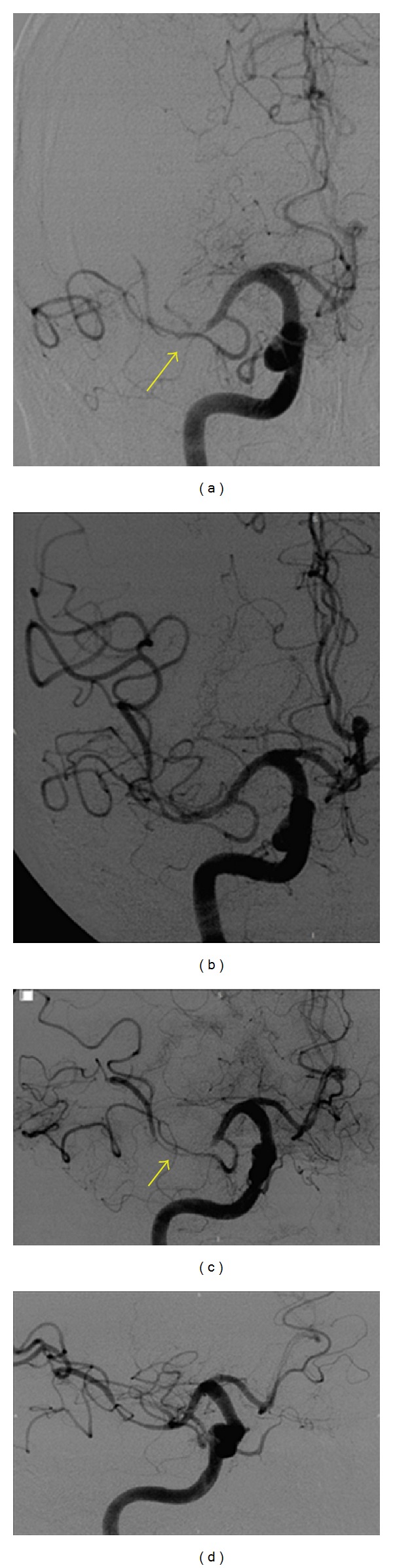
Initial Angiography: anteroposterior view (a) and lateral view (c) evidence complete occlusion of MCA (arrow). Angiography after thrombectomy: anteroposterior view (b) and lateral view (d) show permeable MCA.

**Figure 3:**
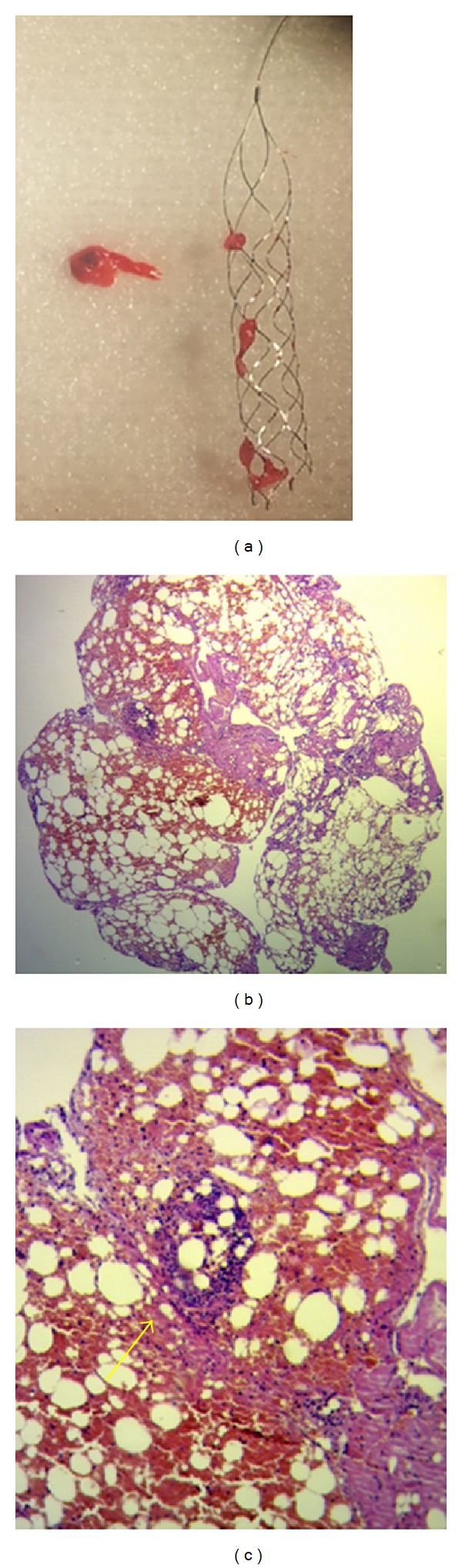
A macroscopic photograph of the fat emboli (a). Histological simple photograph with hematoxylin and eosin at 4x (b) and 10x (c) where an adipose fragment of bone morrow interspersed with fibrin is seen. In addition image (c) shows a bone marrow lymphoid accumulation (arrow).

We proceeded to perform a digital angiography that showed an amputation of MCA prior to its bifurcation (Figures 2(a) and 2(c)). The artery occlusion was recanalized with a microcatheter, through which thrombectomy was done with a Solitaire AB stent (Covidien, USA), retrieving material that was sent for pathological examination (Figure 3(a)). After this procedure, coplete reperfusion of the MCA and its branches was obtained, achivieng a Grade 3 Thrombolysis in cerebral infarcion score (TICI score). Then the patient was transferred to the adult intensive care unit.

Five hours after being admitted to the AICU an episode of bradycardia and hypotension led to a new CT scan which visualized a frontotemporal bleeding hematoma with midline deviation. Additionally, a transcranial Doppler revealed bilateral symmetrical blood flow in the MCA. In this situation it was decided to maintain close monitoring and careful observation.

When color Doppler transesophageal echocardiography was done, color flow and bubbles passage with Valsalva maneuver evidenced PFO.

Throughout the patient postoperative course, conservative treatment was pursued trying to diminish poststroke effects. Four weeks after surgery, the patient was discharged with remaining hemiparesis, dysarthria, and cognitive problems and deceased 3 months later.

## 3. Discussion

The incidence of FES is low (<1%) while FE presents more often [4]. Paradoxical brain FE may occur in patients undergoing hip orthopedic surgery who have an undocumented PFO [5]. Although it has been widely reported, to our knowledge this is the first case report of paradoxical cerebral FE during revision hip surgery.

During hip replacement, acetabular and femur bone preparation, and especially intramedullary instrumentation, cause bone marrow extravasation [7, 8]. Usually fat emboli do not reach systemic circulation as they are retained and filtered in the pulmonary circulation, causing in some cases hypotension, hypoxemia, acute respiratory distress, confusion, coma, or even death [9–11]. Paradoxical embolism occurs when an emboli passes from the pulmonary circulation to the systemic circulation by either a PFO or pulmonary capillaries [9–12].

In a trial done in 1000 consecutive patients with contrast transesophageal echocardiography, the incidence of PFO was 9.2%, and in the decade between 70 and 79 years the incidence was 6.15% [13]. In another trail done by autopsy of 956 normal hearts, a 27.3% incidence of PFO was found, decreasing progressively with increasing age from 34.3% during the first three decades of life to 25.4% during the fourth to eighth decade. In 98% of cases the FOP had 1 to 10 mm diameter with a tendency to increase the size with increasing age, from average of 3.4 mm in the first decade to 5.8 mm in the tenth decade of life [14]. In our patient, the finding of PFO may explain the presence of paradoxical embolism, particularly in the absence of lung involvement.

Riding et al. [12] have detected intraoperative cerebral emboli, with transoesophageal echocardiography, in 18 of 34 (53%) patients with v-a shunt, whereas in patients without shunt they did not detect any episodes of FE. Although FE was not documented intraoperatively by means of transesophageal or transthoracic echocardiography, paradoxical cerebral embolism had to be suspected, when neurologic symptoms were detected, on the basis of the close temporal association between medullary manipulation and the occurrence of symptoms. There are no firm conclusions about the role of Doppler ultrasound to detect embolism during orthopedic surgery [15].

A CT scan was performed immediately because of rapid access, detecting a well-defined hypointense image at birth of the MCA. When cerebral FE is suspected, CT scan and clinical symptoms are not always diagnostic, while MRI is a more sensitive imaging modality and should be performed instead if possible [16]. The use of MRI should be the first step in the diagnostic algorithm to rule out cerebral FE, as it is more sensitive and consistently shows multiple small, scattered lesions that could be negative with other methods [17].

Spinal anesthesia could increase the risk of right-left shunt by altering the circulation volume and arterial and venous pressure due to spasm and in contrast with general anesthesia, by allowing coughs that increase local pressure [18]. In our case an additional risk could have been the left lateral decubitus position, promoting the FE passage through PFO [8].

While PFO can be documented in adults with transthoracic echocardiogram or even better with a transesophageal echocardiography, surgical treatment or closure by catheterization of a PFO should not be considered preoperatively to arthroplasty because of the rare presentation of cerebral embolism [18].

In immediate postoperative orthopedic surgery, while hypotension, tachycardia, or stupor often results from hypovolemic, anemia, or residual sympathetic blockade, it is necessary to consider the possibility of a FE [2]. Close monitoring of the patient is essential during surgery and in the immediate postoperative care. Although neurologic symptoms of cerebral FE are usually transient and entirely reversible, the condition is usually misdiagnosed and fatal if the treatment is delayed. Paradoxical cerebral embolism following revision hip arthroplasty is rare but potentially catastrophic. Early diagnosis requires high index of suspicion and prevents morbidity and mortality with optimal support and immediate treatment.
